# A Misfortune

**Published:** 1877-09-20

**Authors:** 


A Misfortune.—We are very sorry to hear that Dr. W. A. Greene, of Macon, our able contributor, has recently met with a calamity from fire, by which a valuable library, a large collection of journals, original MS., instruments, etc., the accumulations of twenty years, have been swept away. The pecuniary loss is not less than $3,000, while much that was destroyed cannot be estimated in dollars and cents, because of a character which can never be restored.

